# Supplementary material for: Chemical reprogramming ameliorates cellular hallmarks of aging and extends lifespan
Source: EMBO Mol Med. 2025 Jun 30;17(8):2071–94. doi: 10.1038/s44321-025-00265-9 (PMC12340157; doi:10.1038/s44321-025-00265-9)
Supplement: Supplementary file 1 — Appendix [file 44321_2025_265_MOESM1_ESM.pdf]

Chemical reprogramming ameliorates cellular hallmarks of aging and extends lifespan

Lucas Schoenfeldt[1, 2, 3], Patrick T. Paine[1, 2], Sara Picó[1, 2], Nibrasul H. Kamaludeen M.[1], Grace B. Phelps[1,3], Calida Mrabti[1], Gabriela Desdín-Micó[1], María del Carmen Maza[1], Kevin Perez[1,3 *], Alejandro Ocampo[1,3 *]

TABLE OF CONTENT

p2

Appendix Figure S1. Gene expression analysis of chemical-induced partial reprogramming with 7c treatment.

p3

Appendix Figure S2. Serial dilution of the reprogramming chemicals.

p4

Appendix Figure S3. Reduced 2c cocktail efficiently ameliorates multiple hallmarks of aging.

p5

Appendix Table S1. Table of reprogramming chemicals and respective concentrations used.

p5

Appendix Table S2. Primers set for qRT-PCR.

p6

Appendix Table S3. Statistical analyses exact p-values.

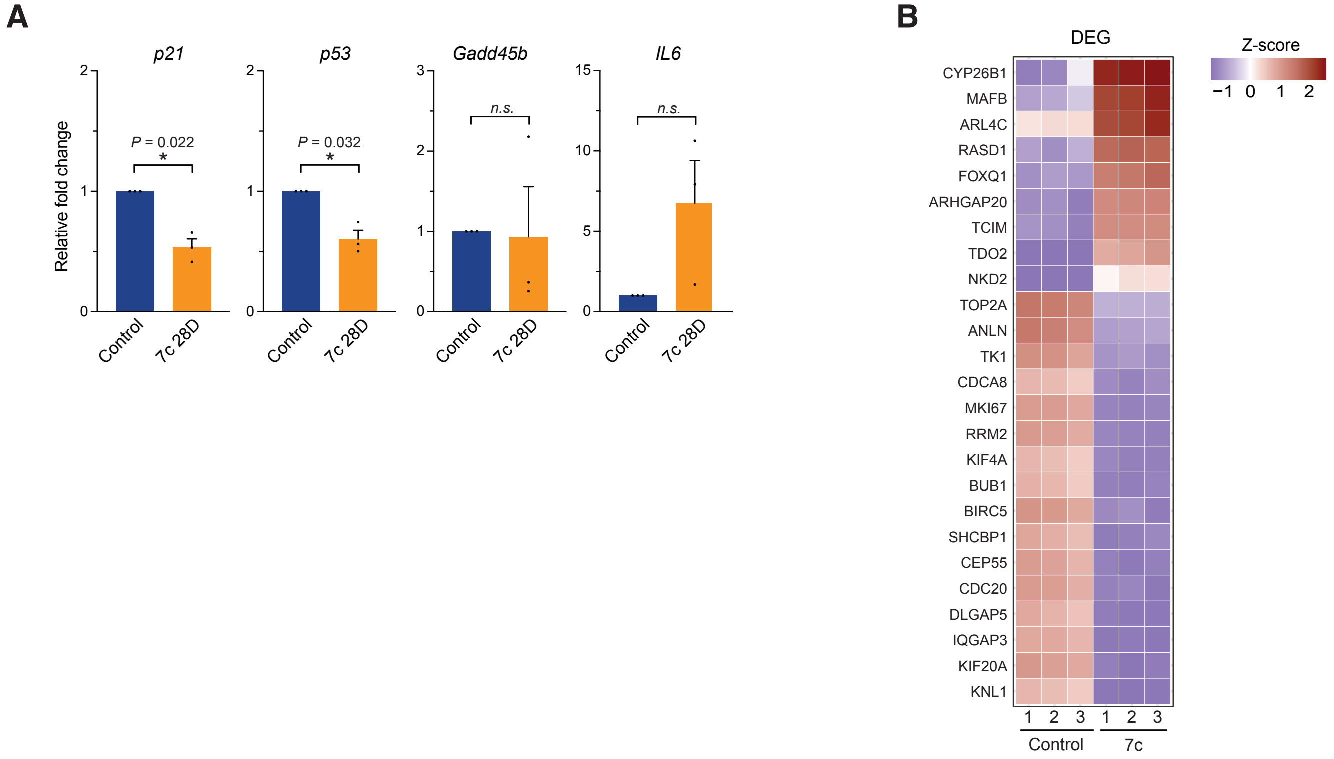

Appendix Figure S1. Gene expression analysis of chemical-induced partial reprogramming with 7c treatment.

(**A**) mRNA levels of senescence-associated and age-related stress response genes in the *p53* tumor suppressor pathway following 7c treatment during replicative-induced senescence (RIS; 28 days). (**B**) Heatmap showing the top differentially expressed genes following 7c treatment relative to untreated controls in human fibroblasts. (**A**,**B**) n=3. DEG, Differentially expressed genes.

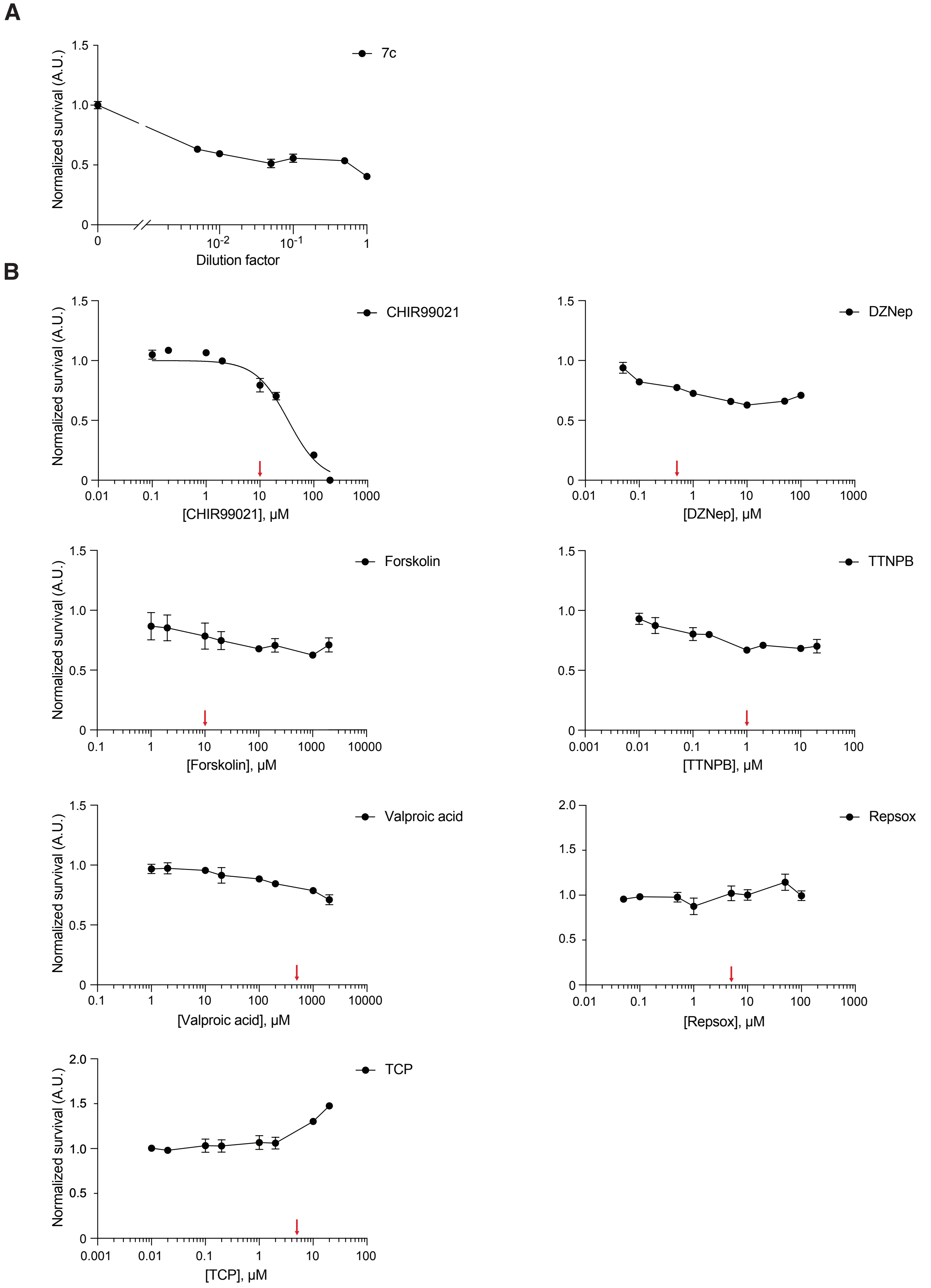

Appendix Figure S2. Serial dilution of the reprogramming chemicals.

(**A**) Crystal violet quantification of cell density following treatment until confluency with serial dilutions of the 7c reprogramming cocktail. (**B**) MTS quantification of cell density following treatment until confluence with different concentrations of the reprogramming chemicals. Red arrows indicate experimental concentrations. Initial concentrations noted in Supplementary Table 2. Nonlinear regression displayed when applicable. Data are mean ± SEM.

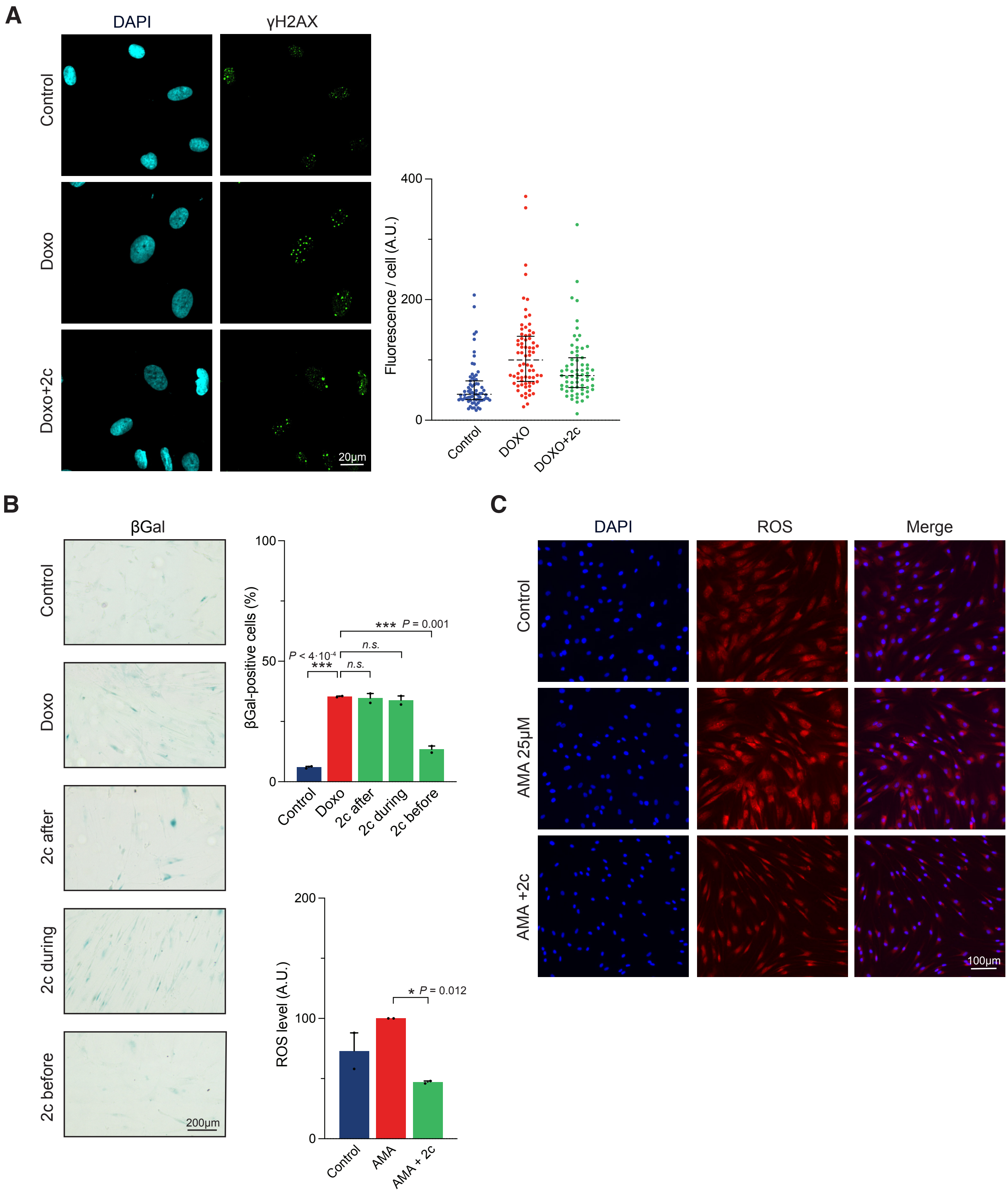

Appendix Figure S3. Reduced 2c cocktail efficiently ameliorates multiple hallmarks of aging.

(**A**) Immunofluorescence and quantification of γH2AX following Doxorubicin (100 nM) and 2c treatment (6 days, "6D"). (**B**) Senescence-associated beta-galactosidase (SA-beta-gal) staining and quantification in 2c treated fibroblasts before, during, and after Doxorubicin (100 nM) treatment. (**C**) Fluorescence detection and quantification of ROS following AMA (25 μM) and 2c treatment. Data are median ± IQR (**A**), mean ± SEM (**B**,**C**). (**A**) n=1, (**B**-**C**) n=2. Statistical significance was assessed by comparison to untreated control using one-way ANOVA and Dunnett correction (**B**), paired two-tailed *t*-test (**C**). AMA, Antimycin A.

Drug	Mol. Weight (kDa)	Concentration used (µM)
Valproic acid	166.2	500
CHIR99021	465.3	10
Repsox	287.3	5
Tranylcypromine	169.7	5
Forskolin	410.5	10
DZNep	298.7	0.5
TTNPB	348.5	1

Appendix Table S1. Table of reprogramming chemicals and respective concentrations used.

Human gene	Sequence (5' → 3')	
18S	Forward	GGCGCCCCCTCGATGCTCTTAG
	Reverse	GCTCGGGCCTGCTTTGAACACTCT
p16	Forward	GGGTCGGGTGAGAGTGG
	Reverse	CGAATAGTTACGGTCGGAGG
p21	Forward	CATGGGTTCTGACGGACATC
	Reverse	TGCCGAAGTCAGTTCCTTGT
p53	Forward	GCTTTCCACGACGGTGAC
	Reverse	GCTCGACGCTAGGATCTGAC
Il6	Forward	AGTGAGGAACAAGCCAGAGC
	Reverse	GTCAGGGGTGGTTATTGCAT
Gadd45b	Forward	ACAGTGGGGGTGTACGAGTC
	Reverse	GATGTCATCCTCCTCCTCCTC
Btg2	Forward	CTCCAGGAGGCACTCACAG
	Reverse	ATGATGGGGTCCATCTTGT

Appendix Table S2. Primers set for qRT-PCR.

Figure panel	Pair	p-value
Fig. 1B	Control-7c	$2.5 \cdot 10^{-10}$
Fig. 1C	Control-Doxo	$1.3 \cdot 10^{-10}$
Fig. 1C	Doxo-Doxo+7c	$1.3 \cdot 10^{-10}$
Fig. 1D	Control-7c	$6.1 \cdot 10^{-36}$
Fig. 1E	Control-7c	$8.7 \cdot 10^{-32}$
Fig. 1F	Control-7c (*Gadd45b*)	$1.6 \cdot 10^{-5}$
Fig. 3A	Control-2c	$1.8 \cdot 10^{-6}$
Fig. 3B	Control-2c	$1.9 \cdot 10^{-28}$
Fig. 3C	Control-2c	$2.0 \cdot 10^{-29}$
Fig. 3G	Control-2c 6D (*p21*)	$2.6 \cdot 10^{-6}$
Fig. 3G	Control-2c 29D (*p21*)	$2.7 \cdot 10^{-6}$
Fig. 4D	DEG 2c-DEG 7c (Upreg.)	$3.1 \cdot 10^{-4}$
Fig. 4D	DEG 2c-DEG 7c (Downreg.)	$7.8 \cdot 10^{-84}$
Fig. 4D	DEG 2c-DEG 7c (All)	$1.3 \cdot 10^{-16}$
Fig. EV1C	Control-2c	$1.1 \cdot 10^{-5}$
Fig. EV1D	Control-2c	$3.2 \cdot 10^{-8}$
Fig. EV1E	Control-2c	$2.2 \cdot 10^{-45}$
Fig. EV1H	Control-Doxo	$8.0 \cdot 10^{-4}$
Fig. EV2A	Control-2c withdrawal	$9.0 \cdot 10^{-4}$
Fig. EV2B	Control-2c withdrawal	$1.1 \cdot 10^{-22}$
Fig. EV2D	Control-7c	$4.1 \cdot 10^{-7}$

Appendix Table S3. Statistical analyses exact p-values.
