# Supplementary material for: Chemical reprogramming ameliorates cellular hallmarks of aging and extends lifespan
Source: EMBO Mol Med. 2025 Jun 30;17(8):2071–94. doi: 10.1038/s44321-025-00265-9 (PMC12340157; doi:10.1038/s44321-025-00265-9)
Supplement: Supplementary file 7 — Expanded View Figures [file 44321_2025_265_MOESM7_ESM.pdf]

Expanded View Figures

Figure EV1. Optimized cocktail (2c) multiparameter rejuvenation of aging hallmarks are recapitulated in human keratinocytes.

(**A**, **B**) MTS quantification of cell density following treatment of human epidermal keratinocyte with TCP (**A**) and Repsox (**B**). Red arrows indicate selected concentrations. (**C**) Immunofluorescence and quantification of γH2AX following 2c treatment in keratinocytes (6 days, "6D"). (**D**, **E**) Immunofluorescence and quantification of H3K9me3 (**D**) and H3K27me3 (**E**) following 2c treatment (6D) in keratinocytes. (**F**) SA-beta-gal staining and quantification of senescence following 2c treatment (6D) in keratinocytes. (**G**, **H**) Immunofluorescence and quantification of γH2AX (**G**) senescence-associated beta-galactosidase (SA-beta-gal) (**H**) following doxorubicin (100 nM) treatment in 2c pretreated keratinocytes (6D). Data were mean ± SEM (**A**, **B**, **F**, **H**), median ± IQR (**C**, **E**, **G**). (**A**–**H**) $n \geq 3$. Statistical significance was assessed by comparison to untreated control using paired two-tailed *t*-test (**C**–**F**), one-way ANOVA and Dunnett correction (**G**, **H**).

© The Author(s)

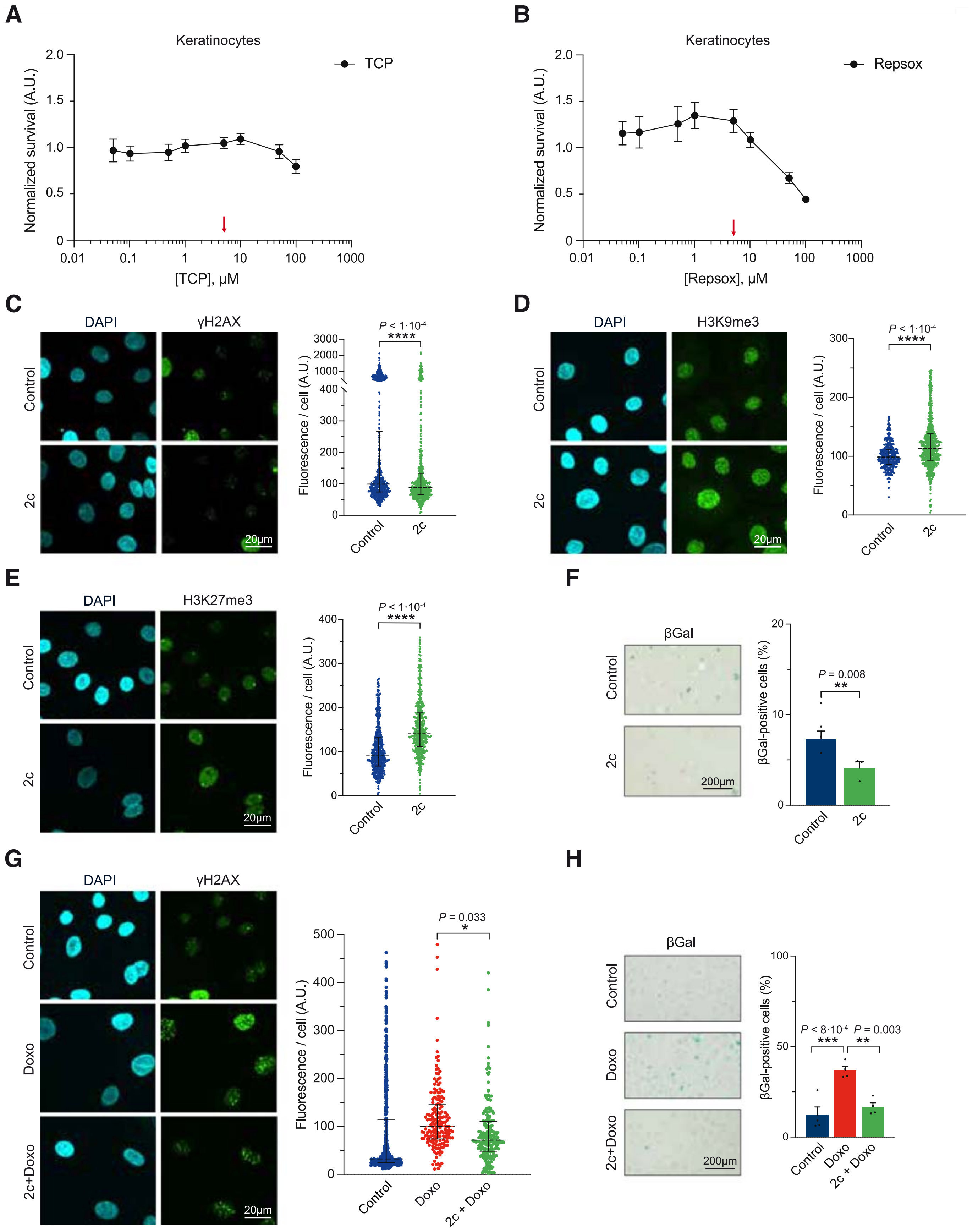
A
Keratinocytes
TCP
Normalized survival (A.U.)
[TCP], μM
B
Keratinocytes
Repsox
Normalized survival (A.U.)
[Repsox], μM
C
DAPI
γH2AX
Control
2c
20μm
P < 1·10-4
****
Fluorescence / cell (A.U.)
D
DAPI
H3K9me3
Control
2c
20μm
P < 1·10-4
****
Fluorescence / cell (A.U.)
E
DAPI
H3K27me3
Control
2c
20μm
P < 1·10-4
****
Fluorescence / cell (A.U.)
F
βGal
Control
2c
200μm
P = 0.008
**
βGal-positive cells (%)
G
DAPI
γH2AX
Control
Doxo
2c+Doxo
20μm
P = 0.033
*
Fluorescence / cell (A.U.)
Control
Doxo
2c + Doxo
H
βGal
Control
Doxo
2c+Doxo
200μm
P < 8·10-4
***
P = 0.003
**
βGal-positive cells (%)
Control
Doxo
2c + Doxo

 © The Author(s)

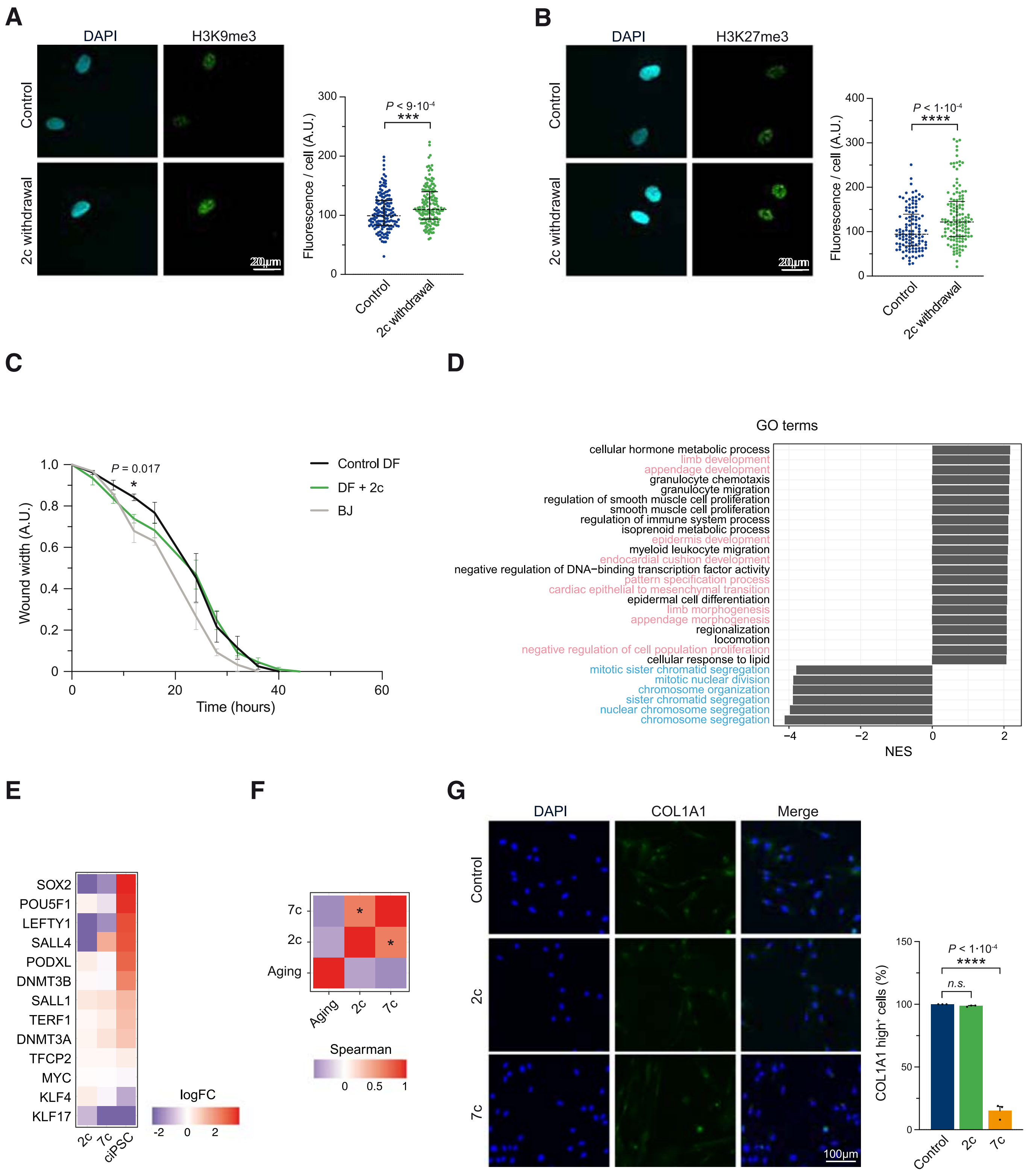
A
DAPI
H3K9me3
Control
2c withdrawal
20µm
Fluorescence / cell (A.U.)
P < 9·10-4
***
Control
2c withdrawal
B
DAPI
H3K27me3
Control
2c withdrawal
20µm
Fluorescence / cell (A.U.)
P < 1·10-4
****
C
P = 0.017
*
Wound width (A.U.)
Time (hours)
Control DF
DF + 2c
BJ
D
GO terms
cellular hormone metabolic process
limb development
appendage development
granulocyte chemotaxis
granulocyte migration
regulation of smooth muscle cell proliferation
smooth muscle cell proliferation
regulation of immune system process
isoprenoid metabolic process
epidermis development
myeloid leukocyte migration
endocardial cushion development
negative regulation of DNA−binding transcription factor activity
pattern specification process
cardiac epithelial to mesenchymal transition
epidermal cell differentiation
limb morphogenesis
appendage morphogenesis
regionalization
locomotion
negative regulation of cell population proliferation
cellular response to lipid
mitotic sister chromatid segregation
mitotic nuclear division
chromosome organization
sister chromatid segregation
nuclear chromosome segregation
chromosome segregation
NES
E
SOX2
POU5F1
LEFTY1
SALL4
PODXL
DNMT3B
SALL1
TERF1
DNMT3A
TFCP2
MYC
KLF4
KLF17
2c
7c
ciPSC
logFC
F
7c
2c
Aging
Spearman
G
DAPI
COL1A1
Merge
Control
2c
7c
100µm
COL1A1 high+ cells (%)
P < 1·10-4
****
n.s.

Figure EV2. Reduced 2c cocktail shows long-lasting effects upon treatment and no dedifferentiation.

(**A**, **B**) Immunofluorescence and quantification of H3K9me3 (**A**) and H3K27me3 (**B**) upon 2c treatment for 6 days (6 days, "6D") followed by 30 days chemical withdrawal. (**C**) Scratch assay analysis upon 2c treatment in adult dermal fibroblasts (DF, 6D) alongside a neonatal control (BJ). (**D**) Gene ontology (GO) terms overlap between 2c and 7c treatments, with developmental (in pink) and cell cycle (in blue) pathways highlighted. (**E**) Heatmap showing the expression pattern of differentially expressed genes associated with pluripotency following 2c and 7c treatment, and induction of chemical iPSC (ciPSC). (**F**) Heatmap of Spearman's correlation of gene expression associated with aging, 2c and 7c. (**G**) Fluorescence detection and quantification of fibroblast identity marker COL1A1 following 2c and 7c treatments. Data were median ± IQR (**A**, **B**), mean ± SEM (**C**, **E**). (**A–G**) $n \geq 3$. Statistical significance was assessed by comparison to untreated control using paired two-tailed *t*-test (**A**, **B**), two-way ANOVA mixed-effects analysis and Geisser-Greenhouse correction (**C**), Spearman's correlation test (**F**), one-way ANOVA and Dunnett correction (**G**). NES normalized enrichment scores, FC fold change.

© The Author(s)

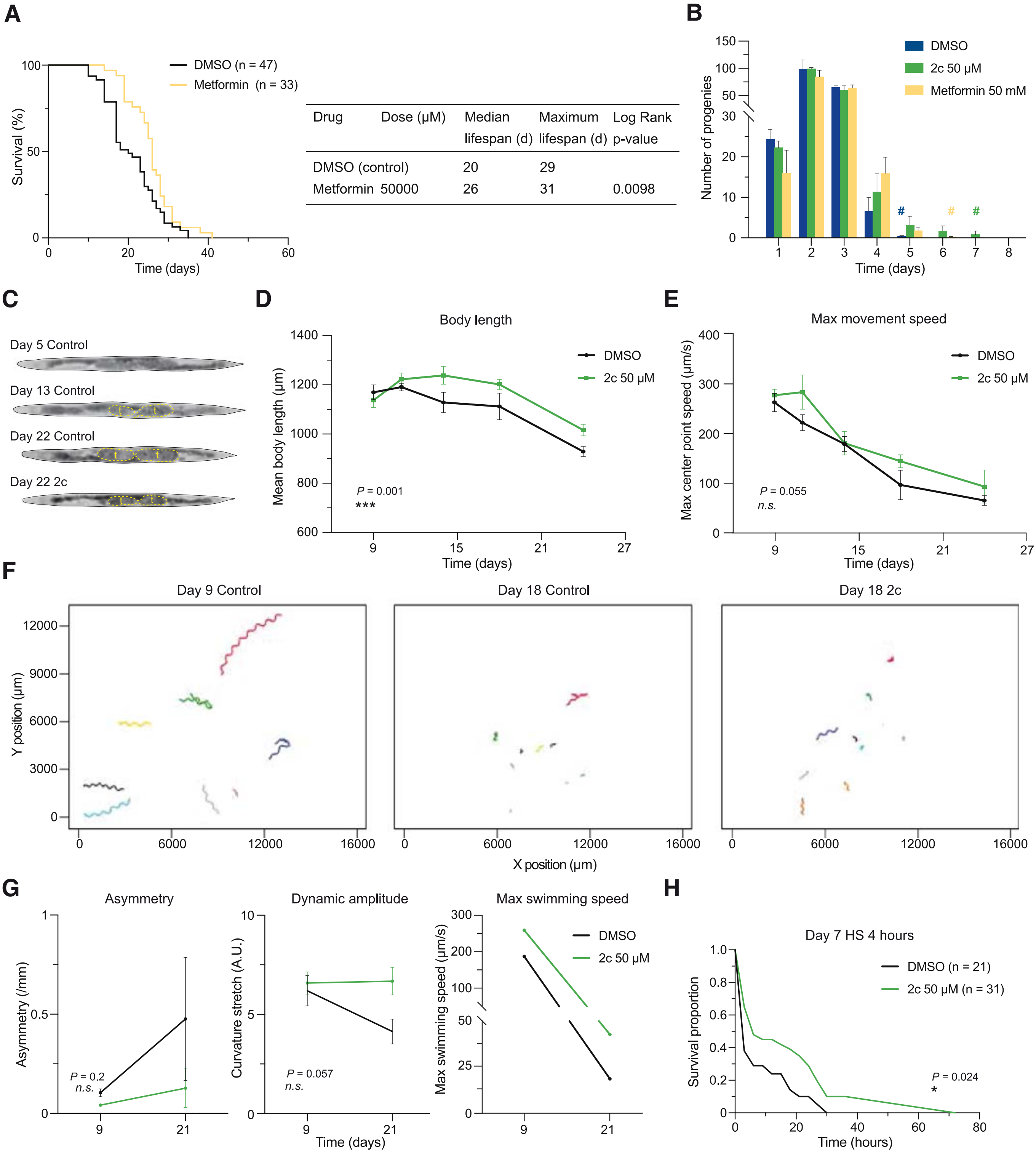

Drug	Dose (μM)	Median lifespan (d)	Maximum lifespan (d)	Log Rank p-value
DMSO (control)		20	29	
Metformin	50000	26	31	0.0098

© The Author(s)

Figure EV3. Treatment with 2c improves multiple healthspan parameters in *C. elegans*.

(**A**) Survival of N2 *C. elegans* upon treatment with Metformin (50 mM). (**B**) Progeny production and reproductive span (denoted by #) of unmated hermaphrodite *C. elegans* upon treatment with 2c and Metformin. (**C**) Germline tumoral mass (denoted by t) development at young (day 5), middle (day 13), and old age (day 22) upon 2c treatment in linearized nematodes. (**D**) Mean body length of N2 *C. elegans* upon treatment with 2c at 50 μM. (**E**) Maximum movement speed of N2 *C. elegans* upon treatment with 2c at 50 μM. (**F**) 30-second movement map of N2 *C. elegans* at day 9 and day 18 upon treatment with 2c. (**G**) Mean swimming asymmetry, amplitude, and max speed of N2 *C. elegans* upon treatment with 2c at 50 μM. (**H**) Thermotolerance of N2 *C. elegans* to 4 h HS at 7 days of adulthood upon 2c treatment. Data were mean ± SEM (**B**, **D**, **E**, **G**). (**B**, **D**, **E**) $n \geq 10$, (**G**) $n \geq 4$. Number of animals and measures are detailed in Methods. Statistical significance was assessed by comparison to vehicle control using log-rank (Mantel-Cox) test (**A**), by comparison to vehicle control using two-way ANOVA mixed-effects analysis and Geisser-Greenhouse correction (**D**, **E**), paired two-tailed *t*-test for day 21 of adulthood (**G**), log-rank (Mantel-Cox) test (**H**). HS heat shock.

 © The Author(s)
